# Supplementary material for: Competing risk of mortality in association studies of non-fatal events
Source: PLoS One. 2021 Aug 13;16(8):e0255313. doi: 10.1371/journal.pone.0255313 (PMC8362942; doi:10.1371/journal.pone.0255313)
Supplement: Attachment — Submitted filename: ReviewersComments_R1.pdf [file pone.0255313.s001.pdf]

Petra BŮŽKOVÁ, Ph.D.
Department of Biostatistics
University of Washington
buzkova@u.washington.edu
206-897-1962

June 14, 2021

Review Comments to the Author

*Reviewer # 1: This paper compares 2 commonly used time-to-event methods: cause specific and subdistribution hazards models applied to studies of non-fatal events. The topic is important, and the manuscript covers well the main points of interest.*

We thank the reviewer for the encouraging words.

1. *In the Introduction, the author stated that one of the main current issue is related to the misuse of terminology when FG subdistribution methodology is used. I indeed agree with the author on this. However, I could not find in the manuscript, any suggestion from the author on an appropriate use of terminology. It would be great, if such a paragraph with appropriate reporting and terminology can be included in the manuscript.*

   We say in the Discussion section: "In conclusion, we find that considering the population of interest is critical to choosing the correct methodology." We believe this is the crucial part of the message we are trying to convey. We added: In such a population (alive individuals), death does not occur by definition, and therefore should not be considered a competing event.

   To address the terminology, we added two paragraphs near the end of the Discussion section, which show how to correctly report and interpret estimates from the two methodologies.

2. *A diagram depicting the differences in allocation of person risks between the 2 methodologies would be helpful, particularly for the readers without a strong statistical background. The section on incidence rates is particularly difficult to follow. A clear definition including allocation of events, person-years and deaths should accompany that section.*

   We thank the reviewer for this suggestion. In the section of incidence rates, we added a figure (Fig3) with boxplots of time at risk for the CHS participants to visually demonstrate the difference between the 2 methodologies. We believe that together with the table of number of events and total person-years at risk (Table 2), as well as the pie chart at the beginning of the paper with allocation of the relevant events (Fig 1), it will clarify the situation.

3. It will also be useful to add a section on the availability and syntaxes for FG methodology in common statistical softwares such as SAS, Stata, R. This section could also include the appropriate use of terminology for reporting in future publication

   As noted for 1), we have added some discussion of how to use and interpret the methodologies' estimates. We note that the ease of using FG has contributed to its misuse and overuse, as discussed in the introduction, and as such we focus on the interpretation of the estimates, rather than their syntax, which is (too) easily found in the literature.

4. *In the discussion, a point is made that FG subdistribution hazard model is generally recommended for clinical prognostic models. However, there can be some limitations. For example, the author may consider commenting on the impact of increasing population's life expectancy on the validity of prognostic models using competing risk of mortality, particularly that these prognostic models are usually developed in study cohorts collected 2 decades prior to their intended use, when mortality risk was much higher than in the current context.*

   We agree with the reviewer that the increasing life expectancy effects prognostic models using FG methodology, as individuals who died in the study, and thus are kept in the risk set but cured of the primary event, might actually not have died in the current time and be at risk of the primary event.

   We added a comment: A complication for prognostic applications of the FG methodology is that prognostic models are often developed from data collected decades ago, and as such the effects of death being protective of the primary outcome are overestimated because of increasing life expectancy. Individuals who died in the study and were thus cured of the primary event may currently stay alive and be at risk.

*Reviewer # 2: I don't have specific suggestions for the authors. The manuscript looks like a PFD of a paper. Has this been published before?*

We assure the reviewer that this paper has not been published before. The pdf file was generated from our latex file, using the recommended PLOS ONE formatting.
